# Psychometric properties of an instrument measuring communication within and between the professional groups licensed practical nurses and registered nurses in anaesthetic clinics

**DOI:** 10.1186/s12913-019-4805-7

**Published:** 2019-12-10

**Authors:** Maria Randmaa, Maria Engström, Gunilla Mårtensson, Christine Leo Swenne, Hans Högberg

**Affiliations:** 1 Faculty of Health and Occupational Studies, University of Gävle, S-801 76 Gävle, Sweden; 2 Department of Public Health and Caring Sciences, Uppsala University, Uppsala, Sweden; 3 Nursing Department, Medicine and Health College, Lishui University, Lishui, China

**Keywords:** Anaesthetic clinic, Communication, Confirmatory factor analysis, ICU nurse-physician questionnaire, Methodological, Psychometric, Validation

## Abstract

**Background:**

The most common cause of clinical incidents and adverse events in relation to surgery is communication error. There is a shortage of studies on communication between registered nurses and licenced practical nurses as well as of instruments to measure their perception of communication within and between the professional groups. The aim of the present study was to evaluate the psychometric properties of the Swedish version of the adapted ICU Nurse-Physician Questionnaire, designed to also measure communication within and between two professional groups: licensed practical nurses and registered nurses. Specifically, the aim was to examine the instrument’s construct validity using confirmatory factor analysis and its internal consistency using Cronbach’s Alpha.

**Methods:**

A cross-sectional and correlational design was used. The setting was anaesthetic clinics in two Swedish hospitals. A total of 316 questionnaires were delivered during spring 2011, of which 195 were analysed to evaluate the psychometric properties of the questionnaire. Construct validity was assessed using confirmatory factor analysis and internal consistency using Cronbach’s Alpha. To assess items with missing values, we conducted a sensitivity analysis of two sets of data, and to assess the assumption of normally distributed data, we used Bayesian estimation.

**Results:**

The results support the construct validity and internal consistency of the adapted ICU Nurse-Physician Questionnaire. Model fit indices for the confirmative factor analysis were acceptable, and estimated factor loadings were reasonable. There were no large differences between the estimated factor loadings when comparing the two samples, suggesting that items with missing values did not alter the findings. The estimated factor loadings from Bayesian estimation were very similar to the maximum likelihood results. This indicates that confirmative factor analysis using maximum likelihood produced reliable factor loadings. Regarding internal consistency, alpha values ranged from 0.72 to 0.82.

**Conclusions:**

The tests of the adapted ICU Nurse-Physician Questionnaire indicate acceptable construct validity and internal consistency, both of which need to be further tested in new settings and samples.

**Trial registration:**

Current controlled trials http://www.controlled-trials.com Communication and patient safety in anaesthesia and intensive care. Does implementation of SBAR make any differences? Identifier: ISRCTN37251313, retrospectively registered (assigned 08/11/2012).

## Background

In industrialized countries, 3–16% of hospitalized patients suffer adverse events [1]. A comprehensive medical record review, conducted in 2016 in 60 Swedish hospitals and covering 52,000 admissions, showed that 8% of hospitalized patients suffered adverse events, at an annual cost of 7 billion SEK [2]. The most common cause of low quality in care has been reported to be communication error [3], which is also the most common cause of clinical incidents and adverse events in relation to surgery [4, 5]. Communication is derived from the Latin *communicare*, which means “make something common”. Several internal (individual) barriers to communication have been described, such as culture, language difference, past experience, expectations, status, prejudice, emotion, deafness, and voice level [6]. In healthcare, barriers to communication between nurses and physicians have been described as being related to the existing hierarchy, differences in communication style between the two professions, lack of a consistent structure, and language [7–9]. An error of communication is defined as “missing or wrong information exchange or misinterpretation or misunderstanding” [10] ^(p. 114)^.

In anaesthetic clinics, different professionals work together in teams of varying size under conditions that change frequently [11] and in an environment that is event-driven, time pressured [12] and marked by frequent distractions [13, 14]. In anaesthetic clinics in Sweden, a team can consist of an anaesthesiologist, surgeon, nurse anaesthetist (RNA)/specialist nurse in intensive care/theatre nurse/registered nurse (RN) and licensed practical nurse (LPN), also called enrolled nurse or nurse’s aid. The composition of the team depends on whether the team works in the operating room (OR), post-anaesthesia unit (PACU) or intensive care unit (ICU).

Instruments designed to measure physician-patient communication [15] and nurse-physician communication [16] have been widely studied. An integrative review of nurse-physician communication showed that nurses and physicians were lacking in interpersonal skills. The authors recommended that future studies explore interpersonal communication across all healthcare professionals [17]. As LPNs is a common professional group in Sweden it is essential that communication within the group and between other professional groups is investigated. There is a shortage of studies concerning the communication between nurses and LPNs as well as of instruments to measure their perceptions of the communication within and between the professional groups.

The aim of the present study was to evaluate the psychometric properties of the Swedish version of the adapted ICU Nurse-Physician Questionnaire, which is designed to also measure communication within and between professional groups: licensed practical nurses (LPNs) and registered nurses (RNs)/nurse anaesthetists (RNA)/specialist nurse in intensive care/theatre nurse. Specifically, the aim was to examine the instrument’s construct validity and its internal consistency.

## Methods

### Design

A cross-sectional and correlational design was used. The present study is part of a project examining staff members’ perceptions of relationships and communication across different professions. The data were collected prior to and after implementation of the communication tool Situation-Background-Assessment-Recommendation (SBAR) at an anaesthetic clinic [18], and the present study is based on baseline data.

### The ICU nurse-physician questionnaire

The original *ICU Nurse-Physician Questionnaire* was derived from the Organizational Culture Inventory and showed Cronbach’s alphas greater than 0.60 [19]. A shortened version consists of six sections: Relationships and communication within the ICU, Teamwork and leadership, Perceived effectiveness, Managing disagreements between physicians/managing disagreements between nurses and physicians, Authority, Satisfaction [20]. The creators of the questionnaire had not completed tests for the shorter version. However, earlier studies carried out in the US [21–23], Canada [24] and Japan [25], using parts of the questionnaire, have shown Cronbach’s alpha between 0.51–0.93. In the present study, the short version section one (Relationships and communications within the ICU) adapted for LPNs was used. It consists of five factors: Within-group communication openness (4 items); Between-group communication openness (4 items); Within-group communication accuracy (4 items); Between-group communication accuracy (3 items); Communication timeliness (3 items). Openness, involves how nurse and physician can say what they mean when speaking with each other without fear or misunderstanding. Accuracy, involves the degree to which nurse and physician believe in the accuracy of the information presented to them by the other party. Timeliness, involves the degree that information about patients is related promptly to personnel who need to be informed [19]. Responses to the items are made on a 5-point Likert scale ranging from “Strongly Disagree” to “Strongly Agree”. The negatively worded items are reversed before factor scores are averaged.

### Swedish version of relationships and communications within the ICU

During the project evaluating relationships and communication between different professionals in an anaesthetic clinic, the ICU Nurse-Physician Questionnaire (Short version, section one) was used [18]. Before the study began, permission to use the questionnaire was obtained from the developer. Four of the authors (MR, ME, GM, and CLS) translated the English version into Swedish. A bilingual professional translator subsequently carried out a back translation. Discrepancies between the two versions were thereafter discussed in the research group, and minor changes were made to make the questions understandable to physicians, RNs and LPNs in the context of a Swedish anaesthetic clinic [26]. The original questionnaire was created to address the relationships and communication between physicians and nurses only. As LPNs are a common staff group in Sweden, the questionnaire was adapted to also measure the relationships and communication between nurses and LPNs.

### Context and participants

The setting was an anaesthetic clinic at two hospitals located in central Sweden, in the same county council and sharing the same top management. All LPNs, nurses and physicians working in the ORs, ICUs and PACUs were invited to participate in the study if they had been working at the anaesthetic clinic for the past 6 months and would be continuing their employment. The specialist nurses in intensive care, the RNAs, and the theatre nurses have a protected professional title, indicating that the person holding the title is a registered nurse with a Master’s degree in either specialist nursing-intensive care, specialist nursing-anaesthesia care or specialist nursing-surgery care [27]. A specialist intensive care nurse has the authority to, when instructed, address, evaluate, and judge, e.g., analgesia and sedation. The title of nurse anaesthetist exists in the US, Sweden, Norway, Denmark, and Switzerland. A nurse anaesthetist has the authority to, when instructed, independently induce, maintain and conclude general anaesthesia, with some support from an anaesthesiologist. The specialist nurse in surgery care has the authority to, e.g., independently ensure that hygienic and aseptic standards are met as well as to organize the work associated with the surgery [28]. In Sweden, LPN is the most common profession within healthcare, with about 250,000 practitioners. The LPN is a vocational degree usually obtained after upper secondary education and does not have a protected professional title. At the anaesthetic clinic, the LPNs work in the OR, PACU and ICU.

### Procedure

A total of 316 questionnaires, and two reminders, were sent out during spring 2011. The questionnaires were coded and sent back in prepaid envelopes to the researchers. The researchers worked at a university and had no employment at the hospital. The respondents were not in any dependency position of the researchers. The response rate was 73% (*n* = 230 of 316), whereof 195 respondents were nurses and LPNs (Table 1). Questionnaires measuring the relationships and communication between physicians and nurses were excluded. The 195 questionnaires were analysed to evaluate the psychometric properties of the Swedish version of the adapted ICU Nurse-Physician Questionnaire (Relationships and communications within the ICU).

### Data analysis

#### Statistical methods

Data were analysed with IBM SPSS Statistics version 22.0; for the CFA we used IBM SPSS AMOS 22.0. Construct validity and especially structural validity were assessed using confirmatory factor analysis (CFA).

The sample consists of 195 individuals. Regarding sample size recommendations, we considered the sample to be adequate for CFA [29]. The ratio of our sample size to the number of items was almost 11:1.

Out of the sample of 195 individuals, there were some internal missing values, i.e. individuals with missing observations in parts of the questionnaire. Fourteen individuals had at least one missing value for some items in the questionnaire.

Our analyses were conducted in two-by-two modelling steps – two ways of handling missing data and two methods of estimation. We first deleted the individuals with at least one missing observation on an item, a so-called listwise deletion. The primary purpose of our initial analysis of listwise deletion of data (missing listwise) was to be able to use modification indices and standardized residuals to look for possible modification in specifications of the model [30]. We then repeated the analysis on complete data (missing casewise) for which all available data were used. No individuals with missing observations on items were deleted from the analysis.

The original CFA model (Model 1) is displayed in Additional file 1: Figure S1. To set the scale of the factors, we restricted one of the regression weights from each of the factors to one. Furthermore, we allowed the factors to correlate with each other. Checks for multivariate normality were conducted using the index of multivariate kurtosis and its critical ratio [30]. The models were fitted to the covariance matrix. The modification indices and standardized residuals were then used to obtain evidence of misfit.

**Table 1:** Sample characteristics. Demographic data on staff members who participated

Profession	RNA	ICU nurse	Theatre nurse	RN	LPN
Total numbers of participants, n	40	69	28	4	54
Sex male/female, n	14/16	5/64	3/25	0/4	0/54
Age					
Md (Q_1_-Q_3_)	46 (40–54)	50 (40–57)	50 (39–52)	44 (34–57)	52 (44–55)
Mean (SD)	47 (9)	48 (10)	47 (7)	45 (12)	50 (9)
Years worked at the clinic					
Md (Q_1_-Q_3_)	11 (3–22)	13 (4–24)	12 (4–27)	8 (2–18)	14 (4–27)
Mean (SD)	13 (10)	15 (11)	15 (11)	9 (8)	16 (11)
Years worked in the profession					
Md (Q_1_-Q_3_)	10 (3–18)	14 (9–24)	25 (8–28)	11 (9–18)	25 (17–31)
Mean (SD)	12 (10)	16 (11)	20 (11)	13 (5)	23 (11)
Overall years worked in healthcare					
Md (Q_1_-Q_3_)	26 (16–34)	29 (19–35)	28 (20–33)	21 (9–38)	31 (22–35)
Mean (SD)	25 (10)	26 (11)	25 (9)	23 (16)	31 (10)

*Md* Median, *Q* Quartiles, *SD* Standard deviation, *n* numbers, *RNA* Nurse anaesthetist, *ICU nurse* Specialist nurse in intensive care, *RN* Registered nurse, *LPN* Licensed Practical Nurse

In each of the two missing data scenarios, we first estimated the model using the maximum likelihood (ML) method. CFA based on listwise deletion assumes that the mechanism underlying the missing data can be categorized as *missing completely at random* (MCAR) [30, 31]. Listwise deletion increases standard errors and reduces statistical power. If the missing data pattern cannot be assumed to be MCAR, the estimates may be biased [30, 32]. When there is missing data on items, i.e., missing casewise, AMOS uses the full information maximum likelihood (FIML) method, which produces consistent and unbiased estimates. Because it uses all information in the data, the method is likely to result in greater statistical power.

Given that responses to our items were made on a Likert scale, we have strictly ordinal scaled categorical data. According to Byrne [30], the methodological approach to the analysis of categorical variables in AMOS is Bayesian estimation. We therefore also conducted the analyses using the Bayesian method of estimation to evaluate estimation diagnostics and to compare the estimates derived from the two estimation methods. Without going into detail, the Bayesian estimation method uses a Markov Chain Monte Carlo (MCMC) algorithm to simulate the posterior distribution from which estimates of the factor loadings are obtained [31]. Other characteristics from the distribution may be used as estimates of standard errors, skewness and kurtosis. The number of simulations needed to obtain stable estimates from the posterior distribution was determined using a convergence statistic [30, 31, 33].

Model fit was assessed using Model Chi^2^, degrees of freedom (df), Chi^2^/df, and goodness-of-fit indices such as comparative fit index (CFI), standardized root mean square residual (SRMR), and root mean square error of approximation (RMSEA) with a *p*-value (PCLOSE) for testing RMSEA no greater than 0.05. The combinations of these fit indices are in line with recommendations in Hooper et.al [34].. Model Chi^2^ is the likelihood ratio test statistic that compares the observed sample covariance matrix with the estimated model covariance matrix. A low value of Model Chi^2^, and in relation to its degrees of freedom, a value of Chi^2^/df < 2.0, is indicative of a well-fitting model. The CFI is based on a comparison of the estimated covariance matrix with the null model in which all variables are uncorrelated, i.e., comparison of the model chi-square to the independence chi-square. According to an earlier recommendation, CFI > 0.90 was indicative of a good fit, but later CFI > 0.95 was suggested [30, 34, 35]. The CFI is thought to perform well with small sample sizes [30, 34]. The SRMR is based on squared differences between the sample correlation matrix and the hypothesized model correlation matrix. Acceptable values for SRMR are lower than 0.08, or preferably lower than 0.05 [30, 34]. Finally, the RMSEA is based on the idea of how well the model, with optimally chosen parameter estimates, would fit the true population covariance matrix. Lower values on this index indicate a better fit. A value below 0.08 indicates good fit, but later recommendations suggest that values below 0.06 indicate good fit [30]. *P*-values (PCLOSE) for testing the null hypothesis RMSEA ≤0.05 should be *p* > 0.05.

The internal consistency of the items was assessed using Cronbach’s alpha coefficient.

The level for statistical significance was set at α = 0.05 for all tests.

## Results

### Construct validity

#### Missing listwise (*n* = 181)

The fit indices for the original model (Model 1) are shown in Table 2, and the model is shown in Additional file 1: Figure S1. According to the criteria for model fit, the indices were not entirely acceptable. Using Modification Indices and Standardized Residuals as well as theoretical reasoning, we tested an modified model (Model 2), Additional file 1: Figure S2, in which the error terms of item ‘ICU9’ (Within-group Communication Accuracy; “I feel that certain ICU nurses [Licensed Practical Nurses] don’t completely understand the information they receive”) and ‘ICU 18’ (Between-group group Communication Accuracy; “I feel that certain ICU nurses [Licensed Practical Nurses] don’t completely understand the information they receive”) were allowed to correlate. The fit indices were improved and now showed a better model fit; see Table 2. In Table 3, the estimates of factor loadings are displayed. Factor loadings from re-estimation of the original model using a Bayesian method are also shown. These factor loadings show only minor discrepancies from the estimates made using the Maximum Likelihood method. Diagnostics from the Bayesian method (see Additional file 2: Table S1) reveal some skewness and kurtosis of the estimates. Moreover, some instability of the estimates was indicated (see Additional file 2: Figure S1a-d of trace and autocorrelation ). This could be a sign of lack of fit in estimating the model using ML, because the items were on an ordinal measurement scale. The critical ratio for multivariate kurtosis for the included items was 11.99 and is a sign of multivariate nonnormality.

**Table 2:** Fit Indices for CFA models (missing listwise data); original model (Model 1) and modified model (Model 2)

Model	CMIN	df	CMIN/df	CFI	SRMR	RMSEA	PCLOSE
1	245.1	125	1.961	0.901	0.077	0.073	0.004
2	224.0	124	1.807	0.918	0.056	0.067	0.027

**Table 3:** Estimates of factor loadings from CFA models (missing listwise data). Model 1 is the original model using Maximum Likelihood method (ML), Model 2 is the modified model (ML) and Bayes Model 1 is Model 1 estimated using the Bayesian method

Factor loadings	Model 1	Model 2	Bayes Model1^a^
ICU 1	0.962	0.962	0.947
ICU 3	1.000	1.000	1.000
ICU 5	0.688	0.690	0.675
ICU 8	0.901	0.904	0.891
ICU 10 RN LPN	1.031	1.031	1.029
ICU 12 RN LPN	1.189	1.190	1.187
ICU 14 RN LPN	1.000	1.000	1.000
ICU 17 RN LPN	1.126	1.125	1.121
ICU 2	0.869	0.820	0.866
ICU 4	1.000	1.000	1.000
ICU 7	0.670	0.654	0.668
ICU 9	0.800	0.787	0.802
ICU 11 RN LPN	1.402	1.361	1.356
ICU 13 RN LPN	1.295	1.267	1.255
ICU 18 RN LPN	1.000	1.000	1.000
ICU 28	0.884	0.884	0.869
ICU 29	1.000	1.000	1.000
ICU 31	0.523	0.523	0.513

^a^The convergence criteria were reached after 500 so-called burn-in samples and an additional 98,500 random draws

#### Missing casewise (*n* = 195)

The fit indices for the original model (Model 1) and the modified model (Model 2) using missing casewise (i.e., using all available data) are shown in Table 4. The fit indices were slightly improved compared to those from the smaller sample and, for the modified model, now showed an acceptable model fit; see Table 4. Table 5 displays the estimates of factor loadings. Factor loadings from a re-estimation using the Bayesian method of the original model are also shown. These factor loadings show only minor discrepancies from the estimates made using the Maximum Likelihood method. Diagnostics from the Bayesian estimation (see Additional file 2) still reveal some skewness and kurtosis of the estimates. Moreover, some instability of the estimates was indicated. Moreover, in the analysis of all available data, this could be a sign of lack of fit in estimating the model using ML because the items were on an ordinal measurement scale. Comparing the estimates of the factor loadings between the two data sets, we found no substantial differences.

**Table 4:** Fit Indices for CFA models missing casewise data; original model (Model 1) and modified model (Model 2)

Model	CMIN	df	CMIN/df	CFI	SRMR^a^	RMSEA	PCLOSE
1	239.4	125	1.915	0.907	NA	0.069	0.012
2	215.5	124	1.738	0.926	NA	0.062	0.083

^a^It is not possible to calculate SRMR when item missing values occur

**Table 5:** Estimates of factor loadings from CFA models (missing casewise data). Model 1 is the original model using Maximum Likelihood method (ML), Model 2 is the modified model (ML) and Bayes Model 1 is Model 1 estimated using the Bayesian method

Factor loadings	Model 1	Model 2	Bayes Model1*
ICU 1	0.915	0.915	0.902
ICU 3	1.000	1.000	1.000
ICU 5	0.647	0.650	0.633
ICU 8	0.885	0.888	0.868
ICU 10 RN LPN	0.983	0.984	0.979
ICU 12 RN LPN	1.139	1.140	1.129
ICU 14 RN LPN	1.000	1.000	1.000
ICU 17 RN LPN	1.118	1.118	1.109
ICU 2	0.907	0.857	0.901
ICU 4	1.000	1.000	1.000
ICU 7	0.685	0.672	0.691
ICU 9	0.834	0.808	0.844
ICU 11 RN LPN	1.451	1.411	1.428
ICU 13 RN LPN	1.341	1.317	1.327
ICU 18 RN LPN	1.000	1.000	1.000
ICU 28	0.882	0.883	0.874
ICU 29	1.000	1.000	1.000
ICU 31	0.539	0.540	0.530

^a^Convergence was achieved after 500 so-called burn-in samples and an additional 98,500 random draws

### Internal consistency

Internal consistency was assessed using Cronbach’s alpha. For each of the five factors, Cronbach’s alpha was above 0.70 (Within-group communication openness 0.78, Between-group communication openness 0.82, Within-group communication accuracy 0.72, Between-group communication accuracy 0.77, and Communication timeliness 0.74).

## Discussion

### Main findings

This is the first study to assess the psychometric properties of the Nurse-Physician Questionnaire (Short version, section one), adapted for LPNs and used in the context of a Swedish anaesthetic clinic. In the study, we found support for the construct validity of the adapted ICU Nurse-Physician Questionnaire as well as for its internal consistency.

Validity often refers to the ability of an instrument to measure what it purports to measure. Finding empirical support for validity is a matter of degree and involves a combination of logical arguments and an ongoing and iterative process. The concept of validity and construct validity has been discussed and defined in various ways [36]. We looked at construct validity as one important component of validity, alongside content validity and criterion validity as in, for example, the COSMIN checklist manual [37]. In the present study, we focused on the structural part of construct validity and used CFA to test whether the a priori structure of the adapted ICU Nurse-Physician Questionnaire fitted the sampled data. This gave partial insight into the scale construct validity. Other aspects of construct validity, such as contrasted groups and hypothesis testing, were not investigated in the study. We used CFA because it is more suitable to testing specific hypotheses regarding the relationship between items and the latent factors than is, e.g., Explorative Factor Analysis (EFA).

The CFA model first tested was a five-factor model with correlated factors and based on cases with complete data, i.e., when cases with at least one missing data point on the items were deleted. This model was not completely satisfactory as judged by the goodness-of-fit indices. A slightly better fit was achieved by letting two error terms correlate. Correlation between the ICU9 – Within-group Communication Accuracy (“I feel that certain ICU [Nurses] don’t completely understand the information they receive”) and the ICU18 – Between-group Communication Accuracy (“I feel that certain ICU [Licensed Practical Nurses] don’t completely understand the information they receive”) was allowed. The correlation was allowed because one can reasonably assume that personnel are unsure as to the extent to which all information is generally understood. Such a correlation may be caused by respondent bias in the associated items or a high degree of overlap in item content [30].

Missing values are a persistent problem in quantitative research. Handling missing values using listwise deletion requires that the mechanism underlying missing values be a completely random one (MCAR) to yield unbiased estimates. This is not a realistic assumption in most cases. Listwise deletion also reduces the number of observations, making the standard error larger. Instead of imputing values, we reanalysed the material using all of the available data. The ML method in AMOS for dealing with missing data is the FIML, which uses all data and produces unbiased, efficient and consistent data, provided we can assume that the missing data are missing at random (MAR), which is a less strict assumption than missing completely at random (MCAR) [31]. Because there were only 14 cases with at least one missing value, the differences between the estimated factor loadings were not particularly large.

Another concern was analysing the ordinal data, as they were quantitative and normally distributed. A common approach is to regard ordinal variables as a crude realization of underlying normally distributed data and to proceed with standard statistical methods or base the CFA on other correlation coefficients, such as polychoric correlations and asymptotic distribution-free methodology requiring very large sample sizes. Many of our variables showed marked skewness and kurtosis, and the index of multivariate kurtosis and its critical ratio indicated multivariate kurtosis. This could lead to biased factor loadings and correlations as well as to too low standard error estimates when using ML estimation [30].

A strength of our study was the use of Bayesian estimation as an alternative to ML estimation for ordinal data. In a Bayesian framework inferences do not depend on normally distributed variables and large samples assumption. Although this framework is quite different in its inference perspective, it is useful for comparison with ML estimates. With normally distributed data and in large samples with no outliers and no missing data, one should expect the estimates in CFA to be close [38]. Despite some skewness, kurtosis and signs of autocorrelations of the estimated factor loadings, they are very similar to the maximum likelihood results. This indicates that CFA using ML produces quite reliable factor loadings, but inflated goodness-of-fit statistics (chi-square values). This information enhances the conclusions from the CFA regarding the validity of the proposed construct.

Explicit calculation of sample size for a given power or precision has not been carried out. Although not satisfactory, we have relied on commonly used recommendations for sample sizes appropriate for CFA. Such recommendations suggest that the ratio of sample size to number of items should vary from 3:1 to 15:1, and a minimum size of 200 [29, 39]. Our sample of 195 comes close to fulfilling these recommendations and may be considered adequate.

Interpretation of the goodness-of-fit indices indicated an acceptable model fit. This is not to say that the construct could not be improved as regards validity. It is also a possibility that our indices were inflated via the model chi-square due to departure from assumptions for using ML.

In conclusion, a strong feature of our study was that we used two sets of data, missing listwise data as well as missing casewise data, to compare the robustness of model to missing cases and that we used two estimation methods, ML and Bayesian, to compare the effects of data level on results.

One limitation with translation of a questionnaire from one language to another language is that there are no linguistic or cultural universals, which can guarantee equivalence between texts. However, the risk of misinterpretation should have been minimized as the questionnaire was translated by four authors (MR, ME, GM, and CLS) who have worked as RNs in hospital care and back translated by a bilingual professional translator. One of the authors (CLS) is a Theatre nurse and another author (MR) is an ICU nurse as well as a RNA and have worked at anaesthetic clinics for decades, and are therefore familiar with the context.

Summing up, the relationships and communication within and between different professional groups are crucial to patient safety. With the original version of Nurse-Physician Questionnaire, nurse-physician relationships and communication can be studied. The strength of the present additions to the adapted ICU Nurse-Physician Questionnaire is that the relationships and communication of the entire personnel group can be studied. However, the generalizability of the survey should be tested in other settings. Hopefully, regular measurements can capture potential relationships and communication difficulties within and between all professional groups, thus allowing measures to improve communication, collaboration and teamwork to be taken in time.

## Conclusion

The tests of the adapted ICU Nurse-Physician Questionnaire indicate acceptable construct validity and internal consistency, both of which need to be further tested in new settings and samples. Although use of ML estimation is not strictly correct for ordinally scaled variables, the estimates did not differ substantially from those obtained using the Bayesian method.

Construct validity is one aspect of the validation of an instrument. CFA provides a mean to test construct validity but should be supported by other evidence as well.

## Supplementary information

**Additional file 1.** A schematic illustration of Model 1 and 2.

**Additional file 2.** Detailed information regarding Bayesian estimation.

## Data Availability

Data that can be shared are included in this published article and its supplementary files. Other data are not publicly available due to participants’ confidentiality.

## Abbreviations

AMOS: Statistical software and it stands for Analysis of a Moment Structures
CFA: Confirmatory Factor Analysis
CFI: Comparative Fit Index
COSMIN: COnsensus-based Standards for the selection of health status Measurement INstruments
EFA: Explorative Factor Analysis
FIML: Full Information Maximum Likelihood
ICU: Intensive Care Unit
LPN: Licensed Practical Nurse
MCAR: Missing Completely At Random
MCMC: Markov Chain Monte Carlo
ML: Maximum Likelihood
OR: Operating Room
PACU: post anaesthesia care unit
RMSEA: Root Mean Square Error of Approximation with a *p*-value PCLOSE
RN: Registered Nurse
RNA: Nurse Anaethetist
SBAR: Situation-Background-Assessment-Recommendation
SEK: Swedish krona
SPSS: Statistical software and it stands for Statistical Package for the Social Sciences
SRMR: Standardized Root Mean square Residual
