# Supplementary material for: Psychometric properties of an instrument measuring communication within and between the professional groups licensed practical nurses and registered nurses in anaesthetic clinics
Source: BMC Health Serv Res. 2019 Dec 10;19:950. doi: 10.1186/s12913-019-4805-7 (PMC6905046; doi:10.1186/s12913-019-4805-7)
Supplement: Supplementary file 1 — Additional file 1. A schematic illustration of Model 1 and 2. [file 12913_2019_4805_MOESM1_ESM.docx]

**Additional file 1**

Figure 1. A schematic illustration of the original model - Model 1. Between-Group (BG), Within-Group (WG).

Figure 2. A schematic illustration of Model 2. Between-Group (BG), Within-Group (WG).
