# Supplementary material for: Psychometric properties of an instrument measuring communication within and between the professional groups licensed practical nurses and registered nurses in anaesthetic clinics
Source: BMC Health Serv Res. 2019 Dec 10;19:950. doi: 10.1186/s12913-019-4805-7 (PMC6905046; doi:10.1186/s12913-019-4805-7)
Supplement: Supplementary file 2 — Additional file 2. Detailed information regarding Bayesian estimation. [file 12913_2019_4805_MOESM2_ESM.docx]

**Additional file 2**

Estimates and diagnostics from Bayesian estimation are shown in Table 1 and 2. These estimates and diagnostics show properties of the simulated joint posterior distribution. Figure 1 and 2 illustrate autocorrelation and trace of the simulations. Because of the method used to estimate the joint posterior distribution using MCMC simulation, it is of interest to look at these types of figures. The autocorrelation plot should exhibit a rapid decay to zero correlation. The trace plot should exhibit a rapid up-and-down variation with no obvious trend. Such patterns indicate that the MCMC procedure converges in distribution and that the summary statistics are accurately estimated.

Table 1. Estimates and diagnostics from Bayesian estimation of Model 1, missing listwise data (n=181). Bold text are values with more marked deviations from ML estimates, greater standard deviations, skewness and/or kurtosis, or smaller covariances.

|  | Mean | S.E. | S.D. | C.S. | Skewness | Kurtosis | Min | Max |
| --- | --- | --- | --- | --- | --- | --- | --- | --- |
| **Parameter estimates factor loadings** |  |  |  |  |  |  |  |  |
|  |  |  |  |  |  |  |  |  |
| ICU_1<--WG_Openness | **0.947** | 0.005 | **0.130** | 1.001 | **0.892** | **1.993** | 0.572 | 1.699 |
| ICU_5<--WG_Openness | **0.675** | 0.004 | 0.101 | 1.001 | **0.752** | **1.474** | 0.380 | 1.203 |
| ICU_8<--WG_Openness | 0.891 | 0.005 | 0.124 | 1.001 | 0.705 | **1.230** | 0.437 | 1.521 |
| ICU_10_RN_LPN<--BG_Openness | 1.029 | 0.004 | 0.102 | 1.001 | 0.204 | 0.168 | 0.654 | 1.603 |
| ICU_12_RN_LPN<--BG_Openness | 1.187 | 0.004 | 0.126 | 1.001 | 0.229 | 0.194 | 0.725 | 1.811 |
| ICU_17_RN_LPN<--BG_Openness | 1.121 | 0.004 | 0.112 | 1.001 | 0.149 | 0.094 | 0.710 | 1.643 |
| Reversed_ICU_2<--WG_Accuracy | 0.866 | 0.005 | **0.138** | 1.001 | 0.331 | 0.480 | 0.399 | 1.498 |
| Reversed_ICU_7<--WG_Accuracy | 0.668 | 0.003 | 0.120 | 1.000 | 0.289 | 0.266 | 0.240 | 1.225 |
| Reversed_ICU_9<--WG_Accuracy | 0.802 | 0.005 | **0.148** | 1.001 | 0.339 | 0.207 | 0.270 | 1.416 |
| Reversed_11_RN_LPN<--BG_Accuracy | **1.356** | 0.007 | **0.180** | 1.001 | 0.533 | 0.734 | 0.733 | 2.279 |
| Reversed_13_RN_LPN<--BG_Accuracy | **1.255** | 0.006 | **0.171** | 1.001 | 0.452 | 0.392 | 0.742 | 2.118 |
| ICU_28<--Timeliness | 0.869 | 0.004 | 0.104 | 1.001 | 0.314 | 0.327 | 0.472 | 1.391 |
| ICU_31<--Timeliness | 0.513 | 0.003 | 0.094 | 1.001 | 0.228 | 0.286 | 0.196 | 0.982 |
|  |  |  |  |  |  |  |  |  |
| **Intercepts** |  |  |  |  |  |  |  |  |
|  |  |  |  |  |  |  |  |  |
| ICU_1 | 4.323 | 0.002 | 0.056 | 1.001 | -0.054 | -0.033 | 4.107 | 4.535 |
| ICU_3 | 3.832 | 0.002 | 0.070 | 1.001 | -0.008 | -0.121 | 3.578 | 4.137 |
| ICU_5 | 4.636 | 0.001 | 0.043 | 1.001 | -0.020 | 0.066 | 4.439 | 4.810 |
| ICU_8 | 4.488 | 0.002 | 0.053 | 1.001 | 0.009 | -0.004 | 4.280 | 4.686 |
| ICU_10_RN_LPN | 4.404 | 0.002 | 0.053 | 1.001 | 0.028 | 0.036 | 4.202 | 4.599 |
| ICU_12_RN_LPN | 4.038 | 0.002 | 0.061 | 1.000 | 0.051 | -0.040 | 3.794 | 4.306 |
| ICU_14_RN_LPN | 4.576 | 0.002 | 0.046 | 1.001 | -0.041 | -0.042 | 4.405 | 4.746 |
| ICU_17_RN_LPN | 4.278 | 0.002 | 0.058 | 1.000 | 0.029 | -0.001 | 4.061 | 4.500 |
| Reversed_ICU_2 | 3.459 | 0.003 | 0.083 | 1.000 | -0.003 | -0.130 | 3.162 | 3.765 |
| Reversed_ICU_4 | 3.560 | 0.002 | 0.083 | 1.000 | -0.082 | 0.027 | 3.233 | 3.871 |
| Reversed_ICU_7 | 3.496 | 0.004 | 0.084 | 1.001 | 0.074 | 0.107 | 3.169 | 3.892 |
| Reversed_ICU_9 | 3.107 | 0.003 | 0.086 | 1.001 | 0.013 | 0.040 | 2.771 | 3.449 |
| Reversed_11_RN_LPN | 3.460 | 0.003 | 0.086 | 1.001 | 0.012 | -0.038 | 3.128 | 3.778 |
| Reversed_13_RN_LPN | 3.586 | 0.004 | 0.079 | 1.001 | -0.062 | 0.054 | 3.253 | 3.911 |
| Reversed_18_RN_LPN | 2.991 | 0.003 | 0.087 | 1.001 | 0.076 | 0.088 | 2.653 | 3.312 |
| ICU_28 | 4.252 | 0.003 | 0.064 | 1.001 | -0.027 | 0.029 | 3.935 | 4.511 |
| ICU_29 | 4.028 | 0.002 | 0.066 | 1.001 | -0.030 | 0.065 | 3.776 | 4.280 |
| ICU_31 | 4.361 | 0.002 | 0.062 | 1.001 | 0.010 | -0.030 | 4.111 | 4.604 |
|  |  |  |  |  |  |  |  |  |
| **Covariances** |  |  |  |  |  |  |  |  |
|  |  |  |  |  |  |  |  |  |
| WG_Openness<->BG_Openness | 0.248 | 0.002 | 0.043 | 1.001 | 0.446 | 0.242 | 0.116 | 0.457 |
| WG_Openness<->WG_Accuracy | 0.177 | 0.003 | 0.062 | 1.001 | 0.473 | 0.539 | -0.011 | 0.463 |
| WG_Openness<->BG_Accuracy | 0.143 | 0.003 | 0.051 | 1.002 | 0.481 | 0.324 | -0.011 | 0.374 |
| Timeliness<->WG_Openness | 0.217 | 0.003 | 0.057 | 1.001 | **0.824** | **2.072** | 0.040 | 0.603 |
| BG_Openness<->WG_Accuracy | **0.079** | 0.002 | 0.043 | 1.001 | 0.181 | 0.258 | -0.117 | 0.253 |
| BG_Openness<->BG_Accuracy | 0.143 | 0.002 | 0.039 | 1.002 | 0.431 | 0.465 | 0.017 | 0.334 |
| Timeliness<->BG_Openness | 0.190 | 0.001 | 0.041 | 1.001 | 0.393 | 0.175 | 0.056 | 0.379 |
| WG_Accuracy<->BG_Accuracy | 0.363 | 0.003 | 0.078 | 1.001 | 0.436 | 0.243 | 0.118 | 0.712 |
| Timeliness<->WG_Accuracy | **0.083** | 0.003 | 0.066 | 1.001 | 0.023 | 0.210 | -0.182 | 0.349 |
| Timeliness<->BG_Accuracy | 0.153 | 0.003 | 0.056 | 1.001 | 0.503 | 0.579 | -0.029 | 0.490 |
|  |  |  |  |  |  |  |  |  |
| **Variances** |  |  |  |  |  |  |  |  |
|  |  |  |  |  |  |  |  |  |
| WG_Openness | 0.391 | 0.005 | 0.094 | 1.001 | 0.490 | 0.463 | 0.118 | 0.801 |
| BG_Openness | 0.266 | 0.002 | 0.043 | 1.001 | 0.348 | 0.035 | 0.133 | 0.427 |
| WG_Accuracy | 0.682 | 0.006 | 0.147 | 1.001 | 0.507 | 0.699 | 0.263 | 1.398 |
| BG_Accuracy | 0.506 | 0.006 | 0.125 | 1.001 | 0.691 | **0.912** | 0.161 | 1.111 |
| Timeliness | 0.617 | 0.005 | 0.106 | 1.001 | 0.367 | 0.019 | 0.302 | 1.044 |
| Err1 | 0.268 | 0.002 | 0.040 | 1.001 | 0.352 | 0.323 | 0.117 | 0.432 |
| Err3 | 0.534 | 0.002 | 0.067 | 1.001 | 0.379 | 0.119 | 0.338 | 0.878 |
| Err5 | 0.166 | 0.001 | 0.023 | 1.000 | 0.434 | 0.252 | 0.098 | 0.266 |
| Err8 | 0.245 | 0.001 | 0.034 | 1.001 | 0.339 | 0.204 | 0.113 | 0.396 |
| Err10 | 0.220 | 0.001 | 0.029 | 1.001 | 0.360 | 0.134 | 0.135 | 0.354 |
| Err12 | 0.339 | 0.002 | 0.043 | 1.001 | 0.448 | 0.490 | 0.206 | 0.554 |
| Err14 | 0.127 | 0.001 | 0.020 | 1.001 | 0.332 | 0.241 | 0.066 | 0.220 |
| Err17 | 0.276 | 0.001 | 0.035 | 1.001 | 0.346 | 0.154 | 0.167 | 0.449 |
| Err2 | 0.742 | 0.004 | 0.103 | 1.001 | 0.375 | 0.376 | 0.427 | 1.275 |
| Err4 | 0.538 | 0.003 | 0.099 | 1.000 | 0.136 | -0.003 | 0.201 | 0.897 |
| Err7 | 0.819 | 0.003 | 0.098 | 1.001 | 0.398 | 0.462 | 0.483 | 1.380 |
| Err9 | 0.926 | 0.005 | 0.120 | 1.001 | 0.334 | 0.465 | 0.517 | 1.567 |
| Err11 | 0.467 | 0.004 | 0.090 | 1.001 | 0.168 | 0.220 | 0.133 | 0.904 |
| Err13 | 0.410 | 0.003 | 0.078 | 1.001 | 0.185 | 0.120 | 0.122 | 0.724 |
| Err18 | 0.884 | 0.003 | 0.107 | 1.000 | 0.589 | **0.862** | 0.572 | 1.469 |
| Err28 | 0.281 | 0.002 | 0.053 | 1.001 | 0.042 | 0.037 | 0.091 | 0.480 |
| Err29 | 0.198 | 0.002 | 0.064 | 1.001 | -0.040 | 0.399 | **-0.083** | 0.461 |
| Err31 | 0.564 | 0.002 | 0.064 | 1.000 | 0.448 | 0.769 | 0.370 | 0.948 |

1. **ICU 1**

1. **ICU 5**

1. **ICU 11**

1. **ICU 13**

Figure 1. Autocorrelation plots and trace plots for four (a – d) items in the simulation process in Bayesian estimation. Model 1 missing listwise data, n=181. The selected items are those with more marked deviations from ML estimates, greater standard deviations, skewness and/or kurtosis in Table 1 above. The autocorrelation plots show a rather slow decay, such that after 100 iterations in the sampling process correlations are 0.25 - 0.35 between the first sampled value and the observation 100 iterations later. The trace plots show a rapid up-and-down pattern without any obvious trends, although some spikes up and down occur now and then. (ssk = RN, usk = LPN)

Table 2. Estimates and diagnostics from Bayesian estimation of Model 1, missing casewise data (n=195). Bold text are values with more marked deviations from ML estimates, greater skewness and/or kurtosis, or smaller covariances.

|  | Mean | S.E. | S.D. | C.S. | Skewness | Kurtosis | Min | Max | Name |
| --- | --- | --- | --- | --- | --- | --- | --- | --- | --- |
| **Regression weights** |  |  |  |  |  |  |  |  |  |
|  |  |  |  |  |  |  |  |  |  |
| ICU_1<--WG_Openness | 0.902 | 0.003 | 0.108 | 1.000 | 0.248 | 0.009 | 0.529 | 1.306 |  |
| ICU_5<--WG_Openness | 0.633 | 0.002 | 0.090 | 1.000 | 0.254 | -0.095 | 0.354 | 1.018 |  |
| ICU_8<--WG_Openness | **0.868** | 0.003 | 0.108 | 1.000 | 0.351 | 0.232 | 0.517 | 1.375 |  |
| ICU_10_RN_LPN<--BG_Openness | 0.979 | 0.003 | 0.096 | 1.000 | 0.202 | 0.076 | 0.603 | 1.365 |  |
| ICU_12_RN_LPN<--BG_Openness | 1.129 | 0.004 | 0.126 | 1.000 | 0.179 | 0.011 | 0.672 | 1.699 |  |
| ICU_17_RN_LPN<--BG_Openness | 1.109 | 0.003 | 0.105 | 1.000 | 0.196 | 0.283 | 0.692 | 1.533 |  |
| Reversed_ICU_2<--WG_Accuracy | 0.907 | 0.005 | 0.142 | 1.001 | 0.347 | 0.341 | 0.412 | 1.642 |  |
| Reversed_ICU_7<--WG_Accuracy | 0.691 | 0.004 | 0.115 | 1.001 | 0.278 | 0.230 | 0.250 | 1.191 |  |
| Reversed_ICU_9<--WG_Accuracy | 0.844 | 0.004 | 0.149 | 1.000 | 0.388 | 0.291 | 0.328 | 1.509 |  |
| Reversed_11_RN_LPN<--BG_Accuracy | **1.428** | 0.009 | 0.201 | 1.001 | **0.680** | 0.724 | 0.898 | 2.410 |  |
| Reversed_13_RN_LPN<--BG_Accuracy | 1.327 | 0.007 | 0.190 | 1.001 | **0.713** | **1.039** | 0.730 | 2.224 |  |
| ICU_28<--Timeliness | 0.874 | 0.004 | 0.096 | 1.001 | 0.158 | 0.079 | 0.534 | 1.313 |  |
| ICU_31<--Timeliness | 0.530 | 0.003 | 0.088 | 1.001 | 0.107 | 0.147 | 0.187 | 0.886 |  |
|  |  |  |  |  |  |  |  |  |  |
| **Intercepts** |  |  |  |  |  |  |  |  |  |
|  |  |  |  |  |  |  |  |  |  |
| ICU_1 | 4.334 | 0.002 | 0.056 | 1.001 | -0.075 | 0.124 | 4.105 | 4.555 |  |
| ICU_3 | 3.832 | 0.002 | 0.068 | 1.001 | -0.008 | 0.010 | 3.554 | 4.102 |  |
| ICU_5 | 4.625 | 0.002 | 0.042 | 1.001 | 0.048 | -0.069 | 4.484 | 4.772 |  |
| ICU_8 | 4.490 | 0.002 | 0.051 | 1.000 | 0.076 | 0.006 | 4.277 | 4.687 |  |
| ICU_10_RN_LPN | 4.388 | 0.002 | 0.051 | 1.001 | 0.045 | 0.025 | 4.213 | 4.596 |  |
| ICU_12_RN_LPN | 4.000 | 0.003 | 0.059 | 1.001 | -0.036 | -0.039 | 3.767 | 4.222 |  |
| ICU_14_RN_LPN | 4.561 | 0.002 | 0.046 | 1.001 | 0.016 | 0.183 | 4.348 | 4.740 |  |
| ICU_17_RN_LPN | 4.269 | 0.002 | 0.056 | 1.000 | 0.019 | -0.071 | 4.065 | 4.495 |  |
| Reversed_ICU_2 | 3.466 | 0.002 | 0.080 | 1.000 | 0.033 | -0.004 | 3.129 | 3.770 |  |
| Reversed_ICU_4 | 3.536 | 0.002 | 0.077 | 1.000 | -0.030 | -0.085 | 3.198 | 3.827 |  |
| Reversed_ICU_7 | 3.501 | 0.002 | 0.079 | 1.000 | -0.034 | 0.007 | 3.213 | 3.812 |  |
| Reversed_ICU_9 | 3.094 | 0.003 | 0.084 | 1.000 | 0.027 | -0.030 | 2.749 | 3.401 |  |
| Reversed_11_RN_LPN | 3.441 | 0.001 | 0.084 | 1.000 | -0.039 | 0.023 | 3.104 | 3.758 |  |
| Reversed_13_RN_LPN | 3.572 | 0.002 | 0.078 | 1.000 | 0.043 | -0.042 | 3.271 | 3.862 |  |
| Reversed_18_RN_LPN | 2.993 | 0.002 | 0.086 | 1.000 | 0.007 | 0.042 | 2.628 | 3.337 |  |
| ICU_28 | 4.252 | 0.002 | 0.062 | 1.000 | 0.009 | 0.080 | 4.014 | 4.479 |  |
| ICU_29 | 4.031 | 0.002 | 0.064 | 1.001 | 0.003 | 0.010 | 3.771 | 4.268 |  |
| ICU_31 | 4.365 | 0.003 | 0.062 | 1.001 | -0.078 | 0.007 | 4.113 | 4.622 |  |
|  |  |  |  |  |  |  |  |  |  |
| **Covariances** |  |  |  |  |  |  |  |  |  |
|  |  |  |  |  |  |  |  |  |  |
| WG_Openness<->BG_Openness | 0.242 | 0.001 | 0.040 | 1.000 | 0.322 | -0.027 | 0.119 | 0.436 |  |
| WG_Openness<->WG_Accuracy | 0.173 | 0.002 | 0.053 | 1.001 | 0.329 | 0.150 | 0.004 | 0.391 |  |
| WG_Openness<->BG_Accuracy | 0.127 | 0.002 | 0.043 | 1.001 | 0.515 | **0.771** | -0.023 | 0.375 |  |
| Timeliness<->WG_Openness | 0.217 | 0.002 | 0.050 | 1.001 | 0.361 | 0.170 | 0.068 | 0.425 |  |
| BG_Openness<->WG_Accuracy | **0.075** | 0.001 | 0.041 | 1.001 | 0.191 | 0.068 | -0.073 | 0.259 |  |
| BG_Openness<->BG_Accuracy | 0.142 | 0.002 | 0.037 | 1.001 | 0.384 | 0.207 | 0.020 | 0.320 |  |
| Timeliness<->BG_Openness | 0.194 | 0.001 | 0.040 | 1.000 | 0.336 | 0.105 | 0.063 | 0.377 |  |
| WG_Accuracy<->BG_Accuracy | 0.327 | 0.003 | 0.072 | 1.001 | 0.502 | 0.568 | 0.122 | 0.712 |  |
| Timeliness<->WG_Accuracy | **0.067** | 0.002 | 0.060 | 1.000 | 0.050 | -0.004 | -0.159 | 0.295 |  |
| Timeliness<->BG_Accuracy | 0.151 | 0.002 | 0.049 | 1.001 | 0.415 | 0.286 | -0.001 | 0.367 |  |
|  |  |  |  |  |  |  |  |  |  |
| **Variances** |  |  |  |  |  |  |  |  |  |
|  |  |  |  |  |  |  |  |  |  |
| WG_Openness | 0.400 | 0.002 | 0.086 | 1.000 | 0.481 | 0.209 | 0.152 | 0.803 |  |
| BG_Openness | 0.274 | 0.001 | 0.043 | 1.001 | 0.345 | 0.111 | 0.129 | 0.461 |  |
| WG_Accuracy | 0.659 | 0.006 | 0.135 | 1.001 | 0.289 | -0.080 | 0.243 | 1.255 |  |
| BG_Accuracy | 0.464 | 0.005 | 0.116 | 1.001 | 0.411 | 0.269 | 0.153 | 1.025 |  |
| Timeliness | 0.601 | 0.004 | 0.098 | 1.001 | 0.395 | 0.092 | 0.275 | 0.998 |  |
| Err1 | 0.259 | 0.001 | 0.036 | 1.001 | 0.255 | 0.267 | 0.132 | 0.416 |  |
| Err3 | 0.536 | 0.002 | 0.065 | 1.001 | 0.386 | 0.221 | 0.347 | 0.897 |  |
| Err5 | 0.207 | 0.001 | 0.025 | 1.001 | 0.276 | 0.054 | 0.124 | 0.335 |  |
| Err8 | 0.246 | 0.001 | 0.035 | 1.001 | 0.353 | 0.245 | 0.122 | 0.400 |  |
| Err10 | 0.227 | 0.001 | 0.029 | 1.001 | 0.407 | 0.315 | 0.135 | 0.381 |  |
| Err12 | 0.371 | 0.001 | 0.046 | 1.001 | 0.286 | 0.013 | 0.227 | 0.601 |  |
| Err14 | 0.143 | 0.001 | 0.022 | 1.001 | 0.176 | -0.157 | 0.070 | 0.226 |  |
| Err17 | 0.280 | 0.001 | 0.036 | 1.001 | 0.315 | 0.077 | 0.166 | 0.475 |  |
| Err2 | 0.730 | 0.003 | 0.102 | 1.001 | 0.387 | 0.479 | 0.332 | 1.188 |  |
| Err4 | 0.541 | 0.004 | 0.096 | 1.001 | 0.232 | 0.181 | 0.153 | 0.964 |  |
| Err7 | 0.832 | 0.004 | 0.097 | 1.001 | 0.386 | 0.226 | 0.537 | 1.297 |  |
| Err9 | 0.923 | 0.004 | 0.115 | 1.001 | 0.376 | 0.526 | 0.519 | 1.531 |  |
| Err11 | 0.449 | 0.003 | 0.088 | 1.001 | 0.190 | 0.197 | 0.145 | 0.844 |  |
| Err13 | 0.398 | 0.003 | 0.076 | 1.001 | 0.161 | 0.147 | 0.092 | 0.704 |  |
| Err18 | 0.890 | 0.004 | 0.100 | 1.001 | 0.398 | 0.229 | 0.566 | 1.291 |  |
| Err28 | 0.276 | 0.002 | 0.049 | 1.001 | 0.263 | 0.217 | 0.096 | 0.500 |  |
| Err29 | 0.198 | 0.003 | 0.059 | 1.001 | -0.117 | 0.302 | **-0.065** | 0.434 |  |
| Err31 | 0.535 | 0.003 | 0.058 | 1.001 | 0.437 | 0.707 | 0.351 | 0.909 |  |

1. **ICU 8**

1. **ICU 11**

1. **ICU 13**

Figure 2. Autocorrelation plots and trace plots for three (a – c) items in the simulation process in Bayesian estimation. Model 1 missing casewise deletion, n=195. The selected items are those with more marked deviations from ML estimates, greater skewness and/or kurtosis in Table 2 above. The autocorrelation plots show a rather slow decay, such that after 100 iterations in the sampling process correlations are 0.25 - 0.30 between the first sampled value and the observation 100 iterations later. The trace plots show a rapid up-and-down pattern without any obvious trends, although spikes up and down occur now and then. (ssk = RN, usk = LPN)
